# Supplementary material for: Mechanism of filament formation in UPA-promoted CARD8 and NLRP1 inflammasomes
Source: Nat Commun. 2021 Jan 8;12:189. doi: 10.1038/s41467-020-20320-y (PMC7794386; doi:10.1038/s41467-020-20320-y)
Supplement: Supplementary file 1 — Supplementary Information [file 41467_2020_20320_MOESM1_ESM.pdf]

# Mechanism of filament formation in UPA-promoted CARD8 and NLRP1 Inflammasomes

## Supplementary Information

Hollingsworth*, David*, and Li* *et al.*

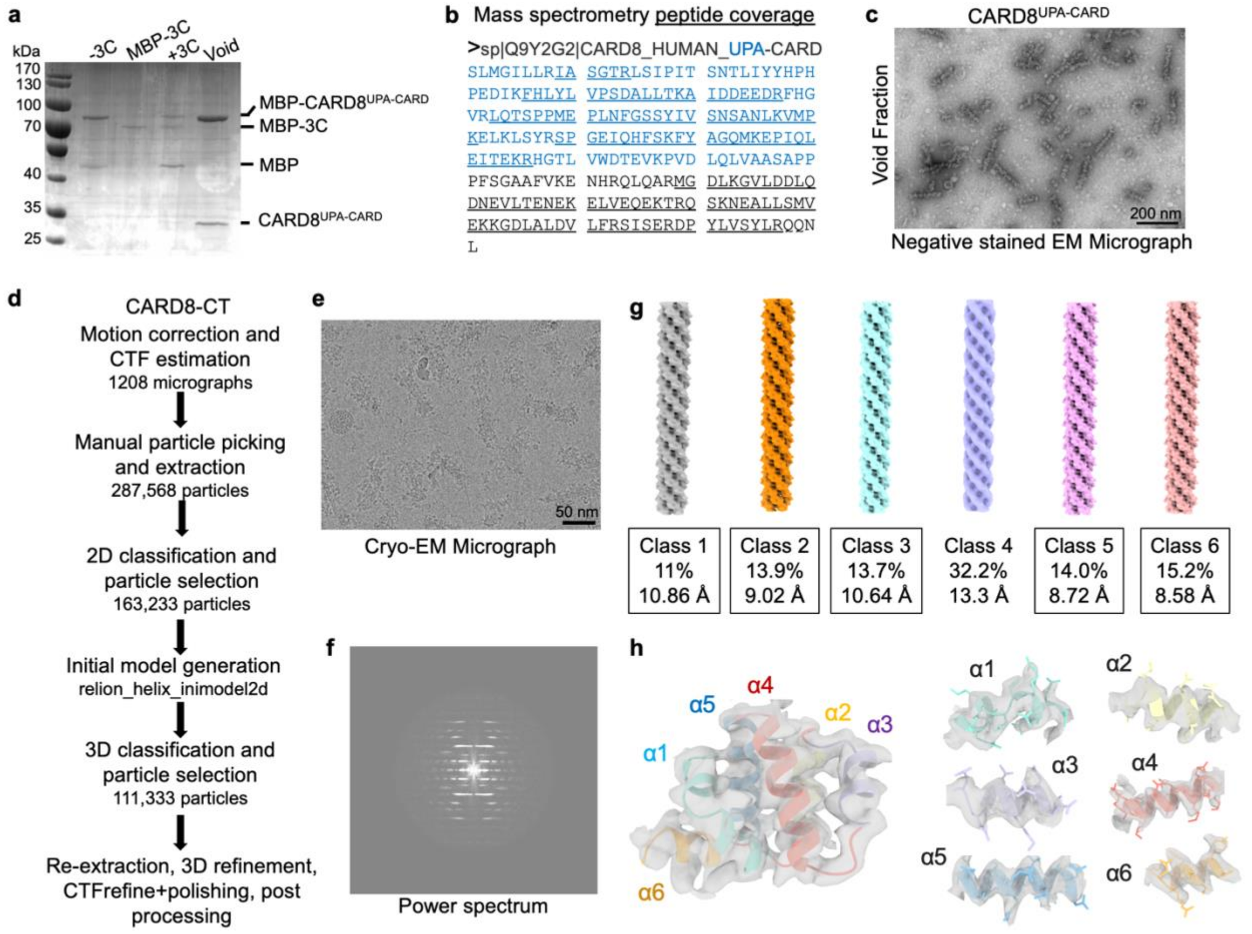


**Supplementary Figure 1 | Structure determination of the CARD8-CT filament.** (**a**) Representative (> 5 independent purifications) SDS-PAGE showing purification of the CARD8$^{UPA-CARD}$ oligomer. Some uncleaved protein incorporates into the growing filaments and is present in the column void fraction. (**b**) Validation of the CARD8$^{UPA-CARD}$ construct by mass spectrometry. Peptides that were detected are underlined. (**c**) Representative (> 5 independent purifications and > 20 fields of view) negative stain EM of the column void fraction showing short CARD-CT filaments. (**d**) Data processing flow chart for structure determination in RELION. (**e**) Representative (> 1000 hand-picked micrographs) cryo-EM micrograph of CARD8-CT filaments. (**f**) Power spectrum derived from a single 2D class average. (**g**) 3D classes generated during data processing. The chosen classes for further refinement are indicated by a box. Each of the six classes is represented by a separate colour. (**h**) Fit of the atomic model of the CARD8$^{CARD}$ core filament into the cryo-EM density. Each of the six helices is represented by a separate colour.

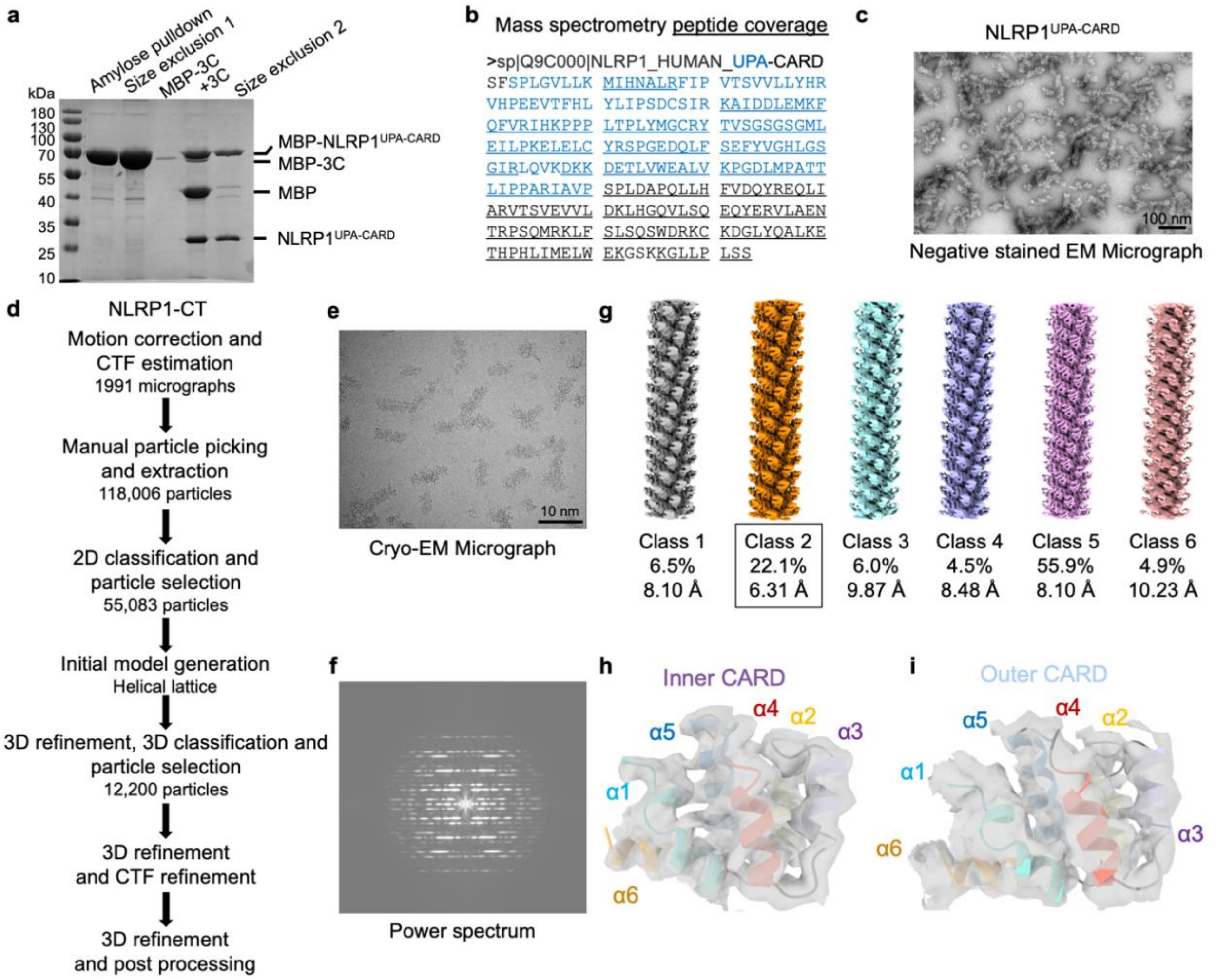


**Supplementary Figure 2 | Structure determination of the NLRP1-CT filament.** (**a**) Representative (> 5 independent purifications) SDS-PAGE showing purification of the NLRP1$^{UPA-CARD}$ oligomer. Some uncleaved protein incorporates into the growing filaments and is present in the column void fraction. (**b**) Validation of the NLRP1$^{UPA-CARD}$ construct by mass spectrometry. Peptides that were detected are underlined. (**c**) Representative (> 5 independent purifications and > 20 fields of view) negative stain EM of the column void fraction showing short NLRP1-CT filaments. (**d**) Data processing flow chart for structure determination in RELION. (**e**) Representative (> 1000 hand-picked micrographs) cryo-EM micrograph of the NLRP1-CT filaments. Representative (**f**) Power spectrum derived from a single 2D class average. (**g**) 3D classes generated during data processing. The chosen classes for further refinement are indicated by a box. Each of the six classes is represented by a separate colour. (**h-i**) Fit of the atomic model of the inner NLRP1$^{CARD}$ (**h**) and the outer NLRP1$^{CARD}$ (**i**) in the cryo-EM density. In (**h-i**), each of the six helices is represented by a separate colour.

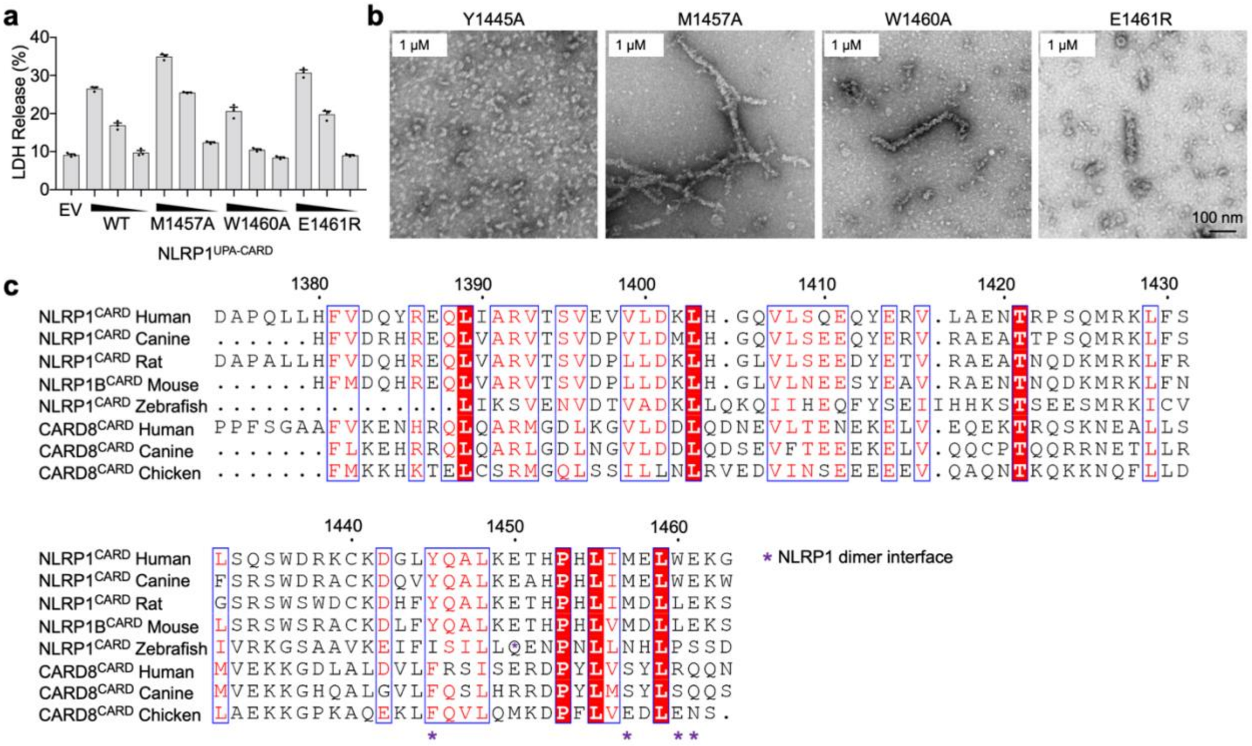


**Supplementary Figure 3 | The NLRP1 dimer interface has limited impact on inflammasome formation.** (**a**) LDH release when NLRP1 UPA-CARD dimer mutants was titrated in HEK293T cells stably expressing caspase-1 and GSDMD, with ASC co-expression. All mutants behaved similarly as the wild-type (WT). EV, empty vector. Data are means ± SEM of n = three biological replicates. (**b**) Filament formation by NLRP1 UPA-CARD dimer mutants in vitro. At the concentration used, most of these mutants formed filaments, like WT (Fig. 3d), except for the Y1445A mutant, which was defective in filament formation, consistent with its impairment in LDH release (Fig. 4d). Filaments formation was performed with two biological replicates. Micrographs are representative of > 5 fields of view. (**c**) Multiple sequence alignment (Clustal Omega) between NLRP1$^{CARD}$ and CARD8$^{CARD}$ homologs, coloured with ESPrint[71]. The NLRP1 dimer interface residues (annotated with purple asterisks) are largely conserved across NLRP1 paralogs in mammals, but not in zebrafish NLRP1 or all CARD8 paralogs.

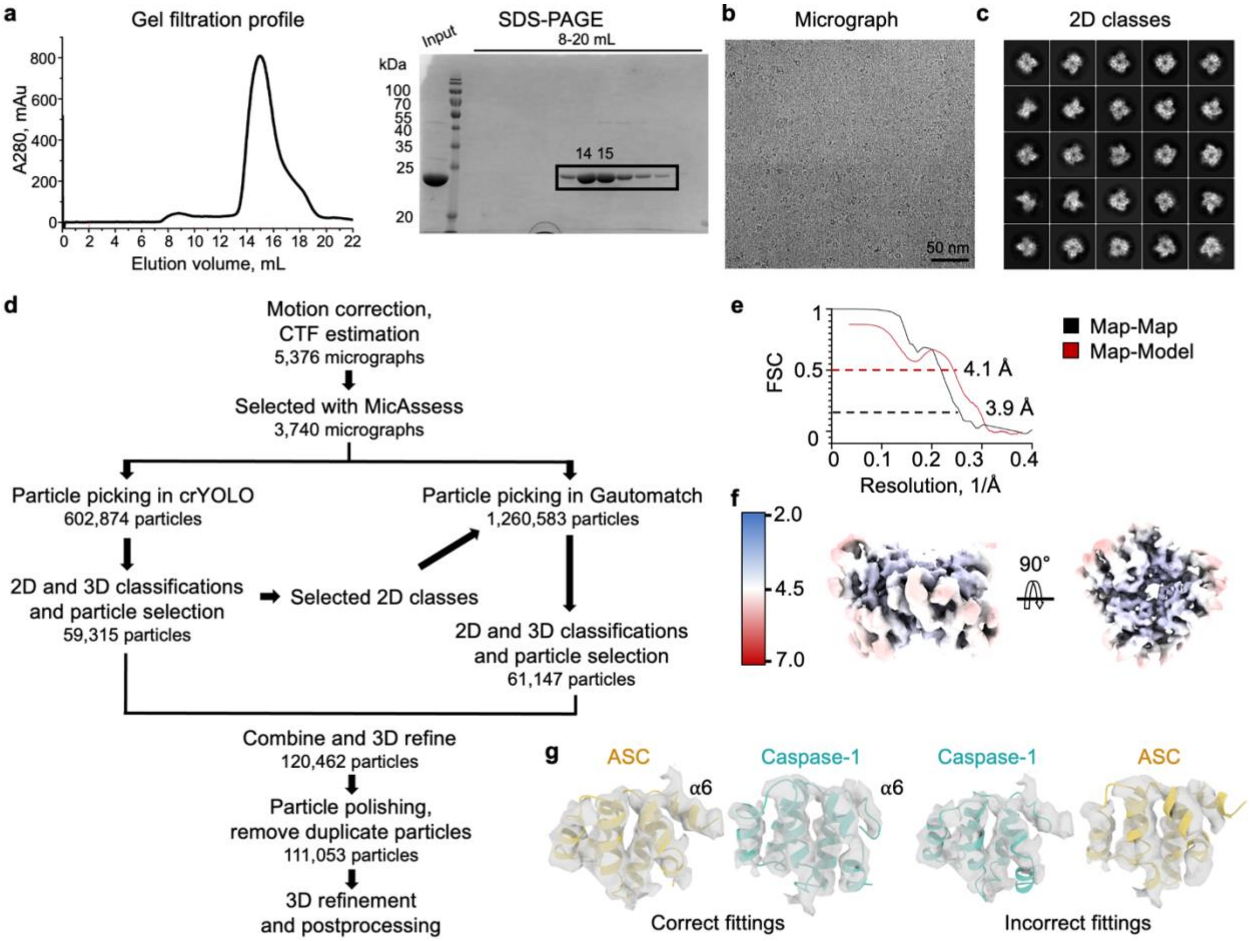


**Supplementary Figure 4 | Structure determination of the ASC-caspase-1 octamer.** (**a**) Purification of the ASC-caspase-1 octamer. The indicated fractions were run on the corresponding SDS-PAGE gel (> 3 independent purifications). (**b**) Representative (> 25 manually inspected) cryo-EM micrograph. (**c**) Representative 2D class averages. (**d**) Data processing flow chart for structure determination in RELION. (**e**) Gold-standard FSC and map-model correlation plots of the ASC-caspase-1 octamer, which gave an overall resolution of 3.9 Å. (**f**) Local resolution of the octamer, calculated with RELION and coloured as indicated. (**g**) Fittings of ASC$^{CARD}$ (brown) and caspase-1$^{CARD}$ (turquoise) into the cryo-EM densities. The incorrectness of the reverse fittings is apparent.

**Supplementary Table 1 | Cryo-EM data collection and refinement statistics**

| | **NLRP1-CT Filament** | **CARD8-CT Filament** | **ASC–caspase-1 Octamer** |
|---|---|---|---|
| **Data collection and processing** | | | |
| Magnification | 81,000 | 96,000 | 105,000 |
| Voltage (kV) | 300 | 300 | 300 |
| Electron exposure (e–/Å$^2$) | 52.3 | 40.0 | 57.12 |
| Defocus range (μm) | −0.8 to −2.2 | −0.8 to −2.2 | −0.8 to −2.5 |
| Physical pixel size (Å) | 1.06 | 0.8315 | 0.825 |
| Symmetry imposed | Helical | Helical | None |
| Initial particle images (no.) | 118,006 | 287,568 | 602,874/1,260,583 |
| Final particle images (no.) | 12,200 | 111,333 | 111,053 |
| Map resolution (Å) at FSC threshold 0.143 | 3.6 | 3.3 | 3.9 |
| | | | |
| **Refinement** | | | |
| Initial model used | 4IFP, 6N1I | 4IKM, 6N1I | 6NIH, 5FNA |
| Model resolution (Å) at FSC threshold 0.5 | 4.2 | 3.5 | 4.1 |
| Map sharpening *B* factor (Å$^2$) | −47.3 | −72.6 | −116.2 |
| **Model composition** | | | |
| Non-hydrogen atoms | 31,276 | 11,056 | 5,306 |
| Protein residues | 3739 | 1360 | 668 |
| **R.m.s. deviations** | | | |
| Bond lengths (Å) | 0.008 | 0.009 | 0.008 |
| Bond angles (°) | 1.282 | 0.812 | 1.809 |
| **Validation** | | | |
| MolProbity score | 1.84 | 1.70 | 3.06 |
| Clashscore | 10 | 12.68 | 42.04 |
| Poor rotamers (%) | 0 | 0 | 5.08 |
| **Ramachandran plot** | | | |
| Favoured (%) | 96.99 | 97.59 | 93.56 |
| Allowed (%) | 3.01 | 2.41 | 5.98 |
| Disallowed (%) | 0 | 0 | 0.46 |

**Supplementary Table 2 | Study primers**

| Construct | Forward (5'-3') | Reverse (5'-3') |
|---|---|---|
| Vector shuttling primers | | |
| pDB-His MBP 3C CARD8 UPA-CARD | aacagcGCTAGCtctctgatgggcatcctgctgcggatc | tagctGCGGCCGCCTAcaaattctgctgtctaagataggacac |
| pDB-His MBP 3C NLRP1 UPA-CARD | gtacaaGCTAGCTCCCCCTTGGGAGTCCTCCTGAAAATGA | tagctGCGGCCGCTCAGCTGCTGAGTGGCA |
| pcDNA3.1 LIC 6B CARD8 UPA-CARD-mCherry | TACTTCCAATCCAATGCCACCATGTCTCTGATGGGCATCCTGCTGC | CTCCCACTACCAATGCCATTCTGCTGTCTAAGATAGGACACGA |
| pcDNA3.1 LIC 6A NLRP1 UPA-CARD-FLAG | TACTTCCAATCCAATGCCACCATGTCCCCCTTGGGAGTCCTCCTGAAAAT | TTATCCACTTCCAATGTTATTACTACTTGTCGTCATCGTCCTTGTAGTCG |
| pDB-His MBP 3C NLRP1 UPA | agttacCATATGTCCCCCTTGGGAGTCCTC | aatcatgCTCGAGCTATGGAGGGATCAGAGTAGTT |
| pDB-His MBP 3C NLRP1 CARD | agttacCATATGCTGCACTTTGTGGACCAG | aatcatgCTCGAGTCAGCTGCTGAGTGGCAGG |
| pDB-His MBP 3C CARD8 CARD | aacagcGCTAGCCCTCCTTTCTCAGGTGCAGCCT | tagctGCGGCCGCCTAcaaattctgctgtctaagataggacac |
| pDB-His MBP ASC-CASP1 | gggaattcCATATGTCGGCAGCCAAGCCAGGCCTGCACTTTATAG | aaggaaaaaaGCGGCCGCTTATGCTGAGAGTCCCAGCGTCCCTGCCAGGTA |
| Mutagenesis primers | | |
| NLRP1 R1392E | GCTGATAGCCgaaGTGACATCGG | TGCTCTCGATACTGGTCC |
| NLRP1 E1397R | GACATCGGTGcggGTTGTCTTGGACAAAC | ACTCGGGCTATCAGCTGC |
| NLRP1 D1401R | GGTTGTCTTGaggAAACTGCATGGACAG | TCCACCGATGTCACTCGG |
| NLRP1 E1411R | GCTGAGCCAGaggCAGTACGAGA | ACCTGTCCATGCAGTTTG |
| NLRP1 E1414R | GGAGCAGTACcggAGGGTGCTGGC | TGGCTCAGCACCTGTCCA |
| NLRP1 R1427E | CAGCCAGATGgagAAGCTGTTCAGCTTG | GGCCTCGTGTTCTCAGCC |
| NLRP1 Y1445A | AGATGGACTCgccCAAGCCCTGAAGG | TTGCACTTCCGGTCCCAG |
| NLRP1 M1457A | TCACCTCATTgccGAACTCTGGGAGAAG | GGATGGGTCTCCTTCAGG |
| NLRP1 W1460A | TATGGAACTCgccGAGAAGGGCAGCAAAAAG | ATGAGGTGAGGATGGGTC |
| NLRP1 E1461R | GGAACTCTGGaggAAGGGCAGCA | ATAATGAGGTGAGGATGGG |
| NLRP1 G1467C | CAGCAAAAAGtgcCTCCTGCCACTC | CCCTTCTCCCAGAGTTCC |
| CARD8 R459E | GGAGAACCACgagCAACTCCAAG | TTCACAAAGGCTGCCTAC |
| CARD8 R464E | ACTCCAAGCCgagATGGGGGACC | TGCCGGTGGTTCTCCTTC |
| CARD8 E485R | TACTGAGAATcggAAGGAGCTGG | AGAACCTCATTGTCTTGG |
| CARD8 E490R | GGAGCTGGTGcggCAGGAAAAGAC | TTCTCATTCTCAGTAAGAACC |

| | | |
|---|---|---|
| CARD8 R495E | GGAAAAGACAgagCAGAGCAAGAATGAGGC | TGCTCCACCAGCTCCTT |
| CARD8 D511K | GAAGAAAGGGaagCTGGCCCTGG | TCCACCATGCTCAGCAAG |
| CARD8 Y527A | AAGGGACCCTgccCTCGTGTCCT | TCACTAATGCTTCTGAAGAGC |
| CARD8 UPA-STOP | AGCCCCTCCTtaaTTCTCAGGTG | GATGCAGCTACAAGCTGG |
| CARD8 A452C | CTCAGGTGCAtgcTTTGTGAAGGAGAAC | AAAGGAGGGCTAGCCATA |
| ASC W169G | CTTCAGTTTCACACCAGCCGGCAACTGGACCTGCAAGGAC | GTCCTTGCAGGTCCAGTTGCCGGCTGGTGTGAAACTGAAG |
| Caspase-1 G20K | CCGTTCCATGGGTGAAAAAACAATAAATGGCTTAC | GTAAGCCATTTATTGTTTTTTCACCCATGGAACGG |
