# Supplementary material for: Mechanism of filament formation in UPA-promoted CARD8 and NLRP1 inflammasomes
Source: Nat Commun. 2021 Jan 8;12:189. doi: 10.1038/s41467-020-20320-y (PMC7794386; doi:10.1038/s41467-020-20320-y)
Supplement: Supplementary file 3 — Reporting summary [file 41467_2020_20320_MOESM3_ESM.pdf]

nature research

Corresponding author(s): Hao Wu

Last updated by author(s): Nov 24, 2020

Reporting Summary

Nature Research wishes to improve the reproducibility of the work that we publish. This form provides structure for consistency and transparency in reporting. For further information on Nature Research policies, see our Editorial Policies and the Editorial Policy Checklist.

Statistics

For all statistical analyses, confirm that the following items are present in the figure legend, table legend, main text, or Methods section.

n/a	Confirmed	
☐	☒	The exact sample size (*n*) for each experimental group/condition, given as a discrete number and unit of measurement
☐	☒	A statement on whether measurements were taken from distinct samples or whether the same sample was measured repeatedly
☐	☒	The statistical test(s) used AND whether they are one- or two-sided<br>*Only common tests should be described solely by name; describe more complex techniques in the Methods section.*
☐	☒	A description of all covariates tested
☒	☐	A description of any assumptions or corrections, such as tests of normality and adjustment for multiple comparisons
☐	☒	A full description of the statistical parameters including central tendency (e.g. means) or other basic estimates (e.g. regression coefficient) AND variation (e.g. standard deviation) or associated estimates of uncertainty (e.g. confidence intervals)
☐	☒	For null hypothesis testing, the test statistic (e.g. *F*, *t*, *r*) with confidence intervals, effect sizes, degrees of freedom and *P* value noted<br>*Give P values as exact values whenever suitable.*
☒	☐	For Bayesian analysis, information on the choice of priors and Markov chain Monte Carlo settings
☒	☐	For hierarchical and complex designs, identification of the appropriate level for tests and full reporting of outcomes
☒	☐	Estimates of effect sizes (e.g. Cohen's *d*, Pearson's *r*), indicating how they were calculated

Our web collection on statistics for biologists contains articles on many of the points above.

Software and code

Policy information about availability of computer code

Data collection	We used SerialEM v3.8.0 and EPU for automated collection of cryo-EM data.
Data analysis	MotionCor2, CTTFFIND4.1, RELION3.1, Gctf v1.06, Coot v0.9, Phenix v1.18.2-3874, refmac v5.7.0032, pymol v2.4.0, UCSF chimera v1.14, UCSF chimeraX v1.0, DeepEMhancer (2020-09-09 install), MicAssess v0.1.0, Fiji v2.1.0/1.53c, PRISM 9, ASTRA V v5.1.2.0, Gautomatch v0.56

For manuscripts utilizing custom algorithms or software that are central to the research but not yet described in published literature, software must be made available to editors and reviewers. We strongly encourage code deposition in a community repository (e.g. GitHub). See the Nature Research guidelines for submitting code & software for further information.

Data

Policy information about availability of data

All manuscripts must include a data availability statement. This statement should provide the following information, where applicable:

- Accession codes, unique identifiers, or web links for publicly available datasets
- A list of figures that have associated raw data
- A description of any restrictions on data availability

Raw cryo-EM data have been deposited in EMPIAR under the accession numbers EMPIAR-10567 [https://dx.doi.org/10.6019/EMPIAR-10567] (CARD8-CT filament), EMPIAR-10564 [https://dx.doi.org/10.6019/EMPIAR-10564] (NLRP1-CT filament), and EMPIAR-10566 [https://dx.doi.org/10.6019/EMPIAR-10566] (ASCCARD-caspase-1CARD octamer). The cryo-EM structures have been deposited in the Electron Microscopy Data Bank (EMDB) with accession numbers EMD-22219 [https://www.ebi.ac.uk/pdbe/entry/emdb/EMD-22219] (CARD8-CT filament), EMD-22220 [https://www.ebi.ac.uk/pdbe/entry/emdb/EMD-22220] (NLRP1-CT filament), and EMDB-22233 [https://www.ebi.ac.uk/pdbe/entry/emdb/EMD-22223] (ASCCARD-caspase-1CARD octamer). The atomic coordinates have been deposited in the protein databank (PDB) with accession numbers 6XKJ [http://doi.org/10.2210/pdb6XKJ/pdb] (CARD8-CT filament), 6XKK [http://doi.org/10.2210/pdb6XKK/pdb] (NLRP1-CT filament), and 7KEU [http://doi.org/10.2210/pdb7KEU/pdb] (ASCCARD-caspase-1CARD octamer). Pymol/chimera session files are available on our Open

Science Framework repository [http://doi.org/10.17605/OSF.IO/X7DV8]. Source data are provided with this paper. Other data are available from the corresponding author upon reasonable request.

Field-specific reporting

Please select the one below that is the best fit for your research. If you are not sure, read the appropriate sections before making your selection.

- [x] Life sciences
- [ ] Behavioural & social sciences
- [ ] Ecological, evolutionary & environmental sciences

For a reference copy of the document with all sections, see nature.com/documents/nr-reporting-summary-flat.pdf

Life sciences study design

All studies must disclose on these points even when the disclosure is negative.

Sample size	No sample size calculations were performed, given the large effect size and/or morphological features of abolishing inflammasome formation. 2-3 biological replicates of each experiment presented are sufficient given the binary outcomes of these experiments, including: negative stain microscopy to determine whether or not structure-guided mutants could form filaments, LDH release to determine inflamamtory cell death, confocal imaging of punctate structures to determine aggregation in cells, and western blotting to show GSDMD/CASP1 cleavage as an indicator of pyroptosis.
Data exclusions	No data were excluded.
Replication	All experiments were confirmed with multiple biological replicates as detailed in Methods or Figure Legends.
Randomization	No randomization was performed, as this does not apply to the in vitro and cellular systems used throughout this study.
Blinding	No blinding was used for imaging, as outcomes were binary (see above).

Reporting for specific materials, systems and methods

We require information from authors about some types of materials, experimental systems and methods used in many studies. Here, indicate whether each material, system or method listed is relevant to your study. If you are not sure if a list item applies to your research, read the appropriate section before selecting a response.

Materials & experimental systems

n/a	Involved in the study	
☐	☒	Antibodies
☐	☒	Eukaryotic cell lines
☒	☐	Palaeontology and archaeology
☒	☐	Animals and other organisms
☒	☐	Human research participants
☒	☐	Clinical data
☒	☐	Dual use research of concern

Methods

n/a	Involved in the study	
☒	☐	ChIP-seq
☒	☐	Flow cytometry
☒	☐	MRI-based neuroimaging

Antibodies

Antibodies used	Caspase-1 (Cell Signaling Technology, #2225, 1:1000), V5 (Abcam, Ab9116, 1:1000), GAPDH (Cell Signaling Technology, 14C10, 1:1000), and GSDMD (Abcam, Ab9116, 1:1000), secondary antibody: IRDye 800CW donkey anti-Rabbit (LI-COR, 926-32213, 1:10,000)
Validation	All antibodies used in these studies have been evaluated by our groups in previously peer-reviewed publications, including in knockout cells. In particular, GSDMD and CASP1 antibodies were validated in knockout cells in Johnson et. al., 2018 Nat Med.

Eukaryotic cell lines

Policy information about cell lines

Cell line source(s)	HEK293T cells stably expressing GSDMD-V5 and caspase-1 were previously described (Johnson et. al, 2018 Nat Med and Ball et. al, Life Sci Alli 2020). Wild-type HEK293T were purchased from the manufacturer (ATCC).
Authentication	Cell lines were purchased and verified by the manufacturer's (ATCC) website and Identity of these cell lines were frequently checked by their morphological features. HEK293T (ATCC) https://www.atcc.org/en/Products/All/CRL-3216.aspx
Mycoplasma contamination	Cell lines regularly tested negative for mycoplasma using the MycoAlert Mycoplasma Detection Kit (Lonza).

Commonly misidentified lines<br>(See ICLAC register)	No commonly misidentified cell lines are used in this study.
